# Biosynthesis of Silver Nanoparticles Using Onion Endophytic Bacterium and Its Antifungal Activity against Rice Pathogen *Magnaporthe oryzae*

**DOI:** 10.3390/jof6040294

**Published:** 2020-11-18

**Authors:** Ezzeldin Ibrahim, Jinyan Luo, Temoor Ahmed, Wenge Wu, Chenqi Yan, Bin Li

**Affiliations:** 1 State Key Laboratory of Rice Biology, Ministry of Agriculture Key Laboratory of Molecular Biology of Crop Pathogens and Insects, Institute of Biotechnology, Zhejiang University, Hangzhou 310058, China; ezzelbehery8818@yahoo.com (E.I.); temoorahmed@zju.edu.cn (T.A.); 2 Department of Vegetable Diseases Research, Plant Pathology Research Institute, Agriculture Research Centre, Giza 12916, Egypt; 3 Department of Plant Quarantine, Shanghai Extension and Service Center of Agriculture Technology, Shanghai 201103, China; toyanzi@126.com; 4 Rice Research Institute, Anhui Academy of Agricultural Sciences, Hefei 230001, China; wuwenge@aaas.org.cn; 5 Institute of Biotechnology, Ningbo Academy of Agricultural Sciences, Ningbo 315040, China

**Keywords:** endophytic bacteria, silver nanoparticles, *Magnaporthe oryzae*, antifungal activity

## Abstract

Biosynthesis of silver nanoparticles (AgNPs) using endophytic bacteria is a safe alternative to the traditional chemical method. The purpose of this research is to biosynthesize AgNPs using endophytic bacterium *Bacillus endophyticus* strain H3 isolated from onion. The biosynthesized AgNPs with sizes from 4.17 to 26.9 nm were confirmed and characterized by various physicochemical techniques such as Fourier transform infrared spectroscopy (FT-IR), X-ray diffraction (XRD), UV-visible spectroscopy, transmission electron microscopy (TEM) and scanning electron microscopy (SEM) in addition to an energy dispersive spectrum (EDS) profile. The biosynthesized AgNPs at a concentration of 40 μg/mL had a strong antifungal activity against rice blast pathogen *Magnaporthe oryzae* with an inhibition rate of 88% in mycelial diameter. Moreover, the biosynthesized AgNPs significantly inhibited spore germination and appressorium formation of *M. oryzae*. Additionally, microscopic observation showed that mycelia morphology was swollen and abnormal when dealing with AgNPs. Overall, the current study revealed that AgNPs could protect rice plants against fungal infections.

## 1. Introduction

Rice (*Oryza sativa* L.) is the largest food crop in the world [1,2]. One of the basic hindrances to the growth of rice crops is the infection of various fungal diseases, particularly rice blast disease caused by *Magnaporthe oryzae*, which poses a serious threat to global food safety through the loss of 10–30% of rice production, enough rice for about 60 million people [1,3,4]. Current control of rice blast disease mainly depends on the use of fungicides. However, the desired goal of controlling the disease has not so far been achieved and there are serious consequences of the excessive use of fungicide on humans, ecosystems and the production of fungicide-resistant strains [5,6]. For all these risks, it is extremely important to find an alternative way to control rice blast disease. Nanotechnology can revolutionize the agricultural and food industry with new tools such as molecular plant disease management, fast disease revelation and the improvement of plants’ ability to uptake nutrients. Furthermore, nanotechnology can enhance our biological knowledge of a variety of plants and therefore can improve crops or nutritional values as well as develop improved systems to monitor environmental conditions and enhance plants’ ability to uptake nutrients or pesticides [7]. Therefore, the application of nanotechnology in agriculture for the control of plant diseases is a safe and eco-friendly alternative to synthetic chemical fungicides [8,9]. It has been instrumental in suppressing many of the fungal pathogens that attack the plants, causing them huge loss. For example, silver nanoparticles (AgNPs) had a significant effect in suppressing many of the air, seed and soil-borne fungal plant pathogens [9,10]. AgNP synthesis was documented using various methods including physical, chemical, and biological [11,12]. However, biological methods are safer than conventional physical and chemical methods [13,14]. It is a simple process (single vessel installation), rapid, cheap and eco-friendly. Furthermore, the polyphenols and various proteins existent in bio-sources work as a reducing agent, decreasing the use of dangerous external chemical reducing agents and, thus, toxicity. The green synthesis procedure does not require any additional capping agents, which further reduces the cost and simplifies the synthetic process. In contrast, chemical and physical methods are highly restricted in large scale applications, and also have high cost, use high energy, waste time and have difficulty in removing waste [15]. One of the most important biological methods to biosynthesize silver nanoparticles is the use of microorganisms such as bacteria, fungi and algae [11]. In this study, for the first time we biosynthesized new AgNPs using cell-free supernatants (CFSs) of endophytic bacterium *B. endophyticus* strain H3 and examined their characterization as a fungicide to inhibit *M. oryzae*.

## 2. Materials and Methods

### 2.1. Microorganisms

The virulent strain Gry of *M. oryzae*, was obtained from the Institute of Biotechnology, Zhejiang University, Hangzhou, China. Strain H3 of the endophytic bacterium isolated from onion plants was used for biosynthesis of AgNPs.

### 2.2. Isolation of Endophytic Bacterium

The entophytic bacterium was isolated from healthy onion plants collected from different locations in Hangzhou, China, according to [16] with slight modification. In brief, onion plants were collected at the seedling stage. The plants were washed carefully with tap water to remove the soil, then dried, and classified into leaves and roots. Onion leaves were cut into small pieces (2–3 cm) and disinfected with alcohol (70%) for 1 min followed by sodium hypoxy-chloride (1%) for 5 min and rinsed three times with sterile water under sterile conditions. One gram of sterile tissues was crushed separately in 9 mL saline water (0.85% NaCl), followed by serial dilution up to 10^−5^. For each of these dilutions, 0.1 mL was spread on nutrient agar (NA) medium [8,11,12] bought from Sangon Biotech, Shanghai, and incubated for two days at 30 °C. The colonies were purified by transferring single colonies to a new NA plate. The isolated strains were stored in 30% (*v*/*v*) glycerol at −80 °C until use.

### 2.3. Identification of Endophytic Bacterium

The isolated endophytic bacterium was identified through 16S rRNA gene sequence analysis. The isolate was grown for 24 h in NA medium and a single colony was transferred to NA broth and incubated in a shaker at 30 °C overnight. Bacterial DNA was isolated using a genomic bacterial DNA isolation Kit (Sangon Biotech (Shanghai) Co, Ltd., Shanghai, China) following the instructions in the protocol. The 16S rRNA gene was amplified by the bacterial-specific primer pairs 27F (AGAGTTTGATCGCTGCTCAG) and 1492F (GGTTACCTTGTTACGACTT) [17,18]. Complete volumes of PCR amplification in 50 μL were performed using the Bioer XP Thermal Cycler (Hangzhou Bioer Tech. Co., Ltd., Hangzhou, China) in 2× TSINGKE PCR Master Mix (TsingKe Biotechnology, Beijing, China). PCR parameters including the following cycles: the initial denaturation stage was 95 °C for 5 min and 30 cycles followed, each of which consisted of denaturation at 94 °C for 30 s, annealing at 53 °C for 30 s, and extending at 72 °C for 1 min. The final extension step was carried out for 5 min at 72 °C. PCR amplicons were verified by using Agarose Gel electrophoresis (1%). PCR products were purified by StarPrep Gel Extraction Kit (GeneStar, Beijing, China) and finally submitted for DNA sequencing in TsingKe Biological Technology, Beijing, China. The 16S rRNA gene sequence of the endophytic bacterium was aligned against a reference database using the BLAST server at National Center for Biotechnology Information (NCBI) (http://www.ncbi.nlm.nih.gov). The phylogenetic tree was constructed using the MEGA 6.0 program and the neighbour-joining method [19]. The sequence was deposited in the NCBI database.

### 2.4. Preparation of the Cell-Free Supernatants of Endophytic Bacterium

The cell-free supernatants (CFSs) of the selected endophytic bacterium were prepared according to [20], with slight modification. In brief, *Bacillus endophyticus* strain H3 was inoculated in NA liquid medium and incubated at 30 °C and 200 rpm for 2 days. The CFSs were purified by centrifugation of bacterial culture (approximately ~1 × 10^8^ CFU/mL) at 10,000 rpm, at 4 °C for 20 min, and twice by using filter sterilization 0.22 μm. To rule out possible contamination, 100 μL of CFSs were spread on NA agar for one day. The CFSs were kept at 4 °C till their use in the biosynthesis of AgNPs.

### 2.5. Biosynthesis of AgNPs

Silver nitrate (Cat. no. 10018461; Sinopharm, Shanghai, China) was used to synthesize AgNPs according to [9], with slight modification. Briefly, 10 mL of CFSs were added to 90 mL of 3 mM AgNO_3_ aqueous solution in a 250 mL Erlenmeyer flask and then incubated at 30 °C in a rotary shaker at 200 rpm for 3 d in the dark. As a control, 10 mL of NA broth with the same volume of AgNO_3_ was used. The successful biosynthesis of AgNPs will convert the colour from yellow light to dark brown, which can be determined by using UV–Visible spectrometry (Shimadzu UV-2550 spectrometer) in the wavelength range of 200–800 nm at a resolution of 1 nm. The resulting pellets were collected from AgNPs by centrifugation at 10,000× *g* for 20 min, purified by washing twice with sterile double- distilled water (ddH_2_O) and stored at −80 °C.

### 2.6. Characterization of the Biosynthesized AgNPs

Characterization of the biosynthesized AgNPs was performed by using several techniques. Fourier transform infrared (FTIR) was performed to identify the functional groups of the CFSs responsible for reducing Ag ion to AgNPs; transmission electron microscopy (TEM), scanning electron microscopy (SEM) and energy dispersive spectrum (EDS) as well as X-ray diffraction (XRD) were performed to study the size and morphology and to ensure the presence of silver ion in the resulting pellets of AgNPs.

#### 2.6.1. FTIR

The functional group of the biosynthesized AgNPs was planned by FTIR as described by [14]. Briefly, 1 mg (freeze-dried) of AgNPs powders were blended with KBr (300 mg) and the FTIR were measured with an AVATAR 370 FTIR spectrometer (Thermo Nicolet, MA, USA) at a spectral range of 500–4000 cm.

#### 2.6.2. XRD

The crystalline phase of AgNPs was determined based on XRD analysis, which was carried out on an XPert PRO diffractometer (Holland) with a detector voltage of 45 kV and a current of 40 mA using CuKo radiation as described in the methods of [13].

#### 2.6.3. TEM, SEM and EDS

The structural morphology and AgNP sizes were studied by TEM and SEM observation using a Transmission Electron Microscopy (JEM-1230, JEOL, Akishima, Japan) and Scanning Electron Microscopy (TM-1000, Hitachi, Japan) according to the method of [12]. In short, the AgNPs powder was equipped with a copper-coated grid and a carbon-coated grid, respectively, for one day at room temperature to form a film of the sample. The existence of silver ion was confirmed by energy dispersive spectrum (EDS).

### 2.7. Antifungal Activity of the Biosynthesized AgNPs

#### 2.7.1. Effect of AgNPs on Mycelium Growth

The inhibitory effect of AgNPs at four concentrations (10, 20, 30 and 40 µg/mL) on mycelium growth of *M. oryzae* strain Gry was determined using an agar medium test as described by [9], with slight modification. In brief, a disk (10-mm in diameter) of 7-day-old mycelium was inoculated in the centre of the petri dishes (9 cm in diameter), containing the blend of Potato Dextrose Agar (PDA) medium (pH 7.0), at several concentrations of AgNPs. The PDA plates were used without AgNPs as a control. The diameter of the fungus colony was measured after 7 d of incubation at 27 °C and then the inhibition of mycelium growth was calculated.

#### 2.7.2. Effect of AgNPs on Cell Wall Morphology

Damage to the cell wall of *M. oryzae* strain Gry was determined using AgNPs according to the method of [21], with minor modification. In short, a mycelial disc of *M. oryzae* strain Gry (10-mm in diameter) was brought from PDA medium, treated and not treated by AgNPs, and examined by using both SEM (TM-1000, Hitachi, Japan) and TEM (JEM-1230, JEOL, Akishima, Japan).

#### 2.7.3. Effect of AgNPs on Spore Germination and Length of Germ Tubes

The influence of four concentrations (10, 20, 30 and 40 µg/mL) of AgNPs on the germination of spores and length of germ tubes of *M. oryzae* strain Gry were identified as described by [22], with slight modification. Briefly, 500 μL of spore suspension (1 × 10^5^ spores/mL) that were prepared according to the method of [23], were added to the same volume of AgNPs in 1 mL tubes with a final concentration of 10, 20, 30 and 40 µg/mL. Mixed spore suspensions with ddH_2_O were used as the control. The germination rate of spores and length of germ tubes of *M. oryzae* strain Gry was recorded after 24 h of incubation at 28 °C in the dark, using the light microscope. The experiment was repeated twice with three replicates.

#### 2.7.4. Effect of AgNPs on Appressorium Formation

The influence of four concentrations (10, 20, 30 and 40 µg/mL) of AgNPs on the appressorium formation of *M. oryzae* strain Gry was assessed according to [6], with slight modification. In brief, 500 μL of spore suspension (1 × 10^5^ spores/mL) was added to the same volume of AgNPs in 1 mL tubes with a final concentration of 10, 20, 30 and 40 µg/mL. Spore suspensions with ddH_2_O were added as a control. The inhibitory effect of AgNPs on numbers and sizes of appressoria were recorded after 72 h of incubation at 28 °C in the dark, using the light microscope.

### 2.8. Statistical Analysis

All experiments were performed randomly and the results were shown as the mean ± SD (standard deviation). Statistical analysis was performed with the SPSS version software package 16.0 (SPSS Inc., Chicago, IL, USA). Differences between the groups were estimated using different test analyses. When the *p* value is < 0.05 or 0.01, the result is statistically significant.

## 3. Results and Discussion

### 3.1. Isolation and Identification of the Endophytic Bacterium

**Figure 1:**
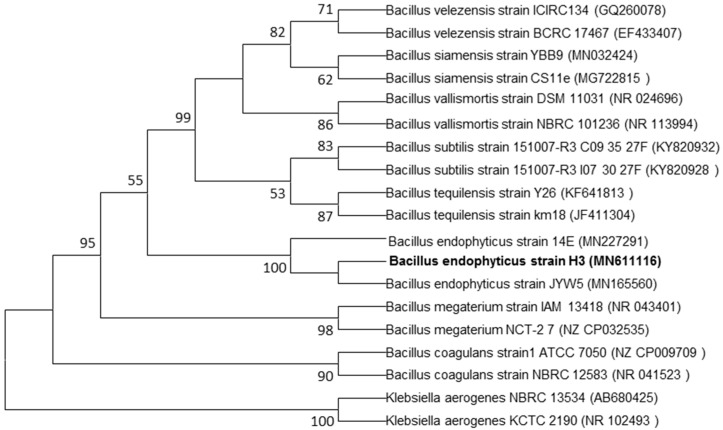
Phylogenetic tree of endophytic bacterium *B. endophyticus* strain H3 isolated from onion leaves in the seedling stage constructed by using 16S rRNA gene sequences. Bootstrap analysis (1000 replicates) for node values greater than 50% are given. Bar 0.02 substitutions per nucleotide position.

Endophytic bacteria live inside of plants without causing harm to the host plant [24]. In our study, we obtained a total of 17 endophytic bacterial isolates from the interior of onion leaves. One isolate, namely, H3 was selected based on its ability in the biosynthesis of AgNPs. This isolate was subjected to molecular identification. Partial 16S rRNA gene sequence analysis revealed that the selected isolate was belonging to the genus of *Bacillus* (Figure 1), and subjected to GenBank under accession number MN611116.

### 3.2. Biosynthesis and Characterization of AgNPs

CFSs obtained from endophytic bacterium *B. endophyticus* strain H3 were tested in the biosynthesis of AgNPs in 3 mM of AgNO_3_ solution. Incubation of 10 mL of CFSs with 90 mL of AgNO_3_ for three days resulted in color conversion from light yellow to dark brown, demonstrating the development of nanoparticles in the reaction mix. Differences in the color of AgNPs have been reported due to the formation of the biomolecules responsible for the synthesis of nanoparticles and the reduction of Ag^+^ to Ag^0^ [9,12,13,25]. The reduction of silver ion (Ag^+^) of AgNO_3_ has been found in many bacterial species, such as the *Bacillus siamensis* strain C1 [14] and *Pseudomonas rhodesiae* [13]. During the reduction of AgNO_3_, the nitrate ions (NO_3_^−^) are reduced to nitrite (NO_2_^–^) by firstly accepting two protons and then releasing two electrons and water. The electrons emitted in the reduction reaction are transferred to the Ag^+^ to form the silver element Ag^0^ [26,27].

**Figure 2:**
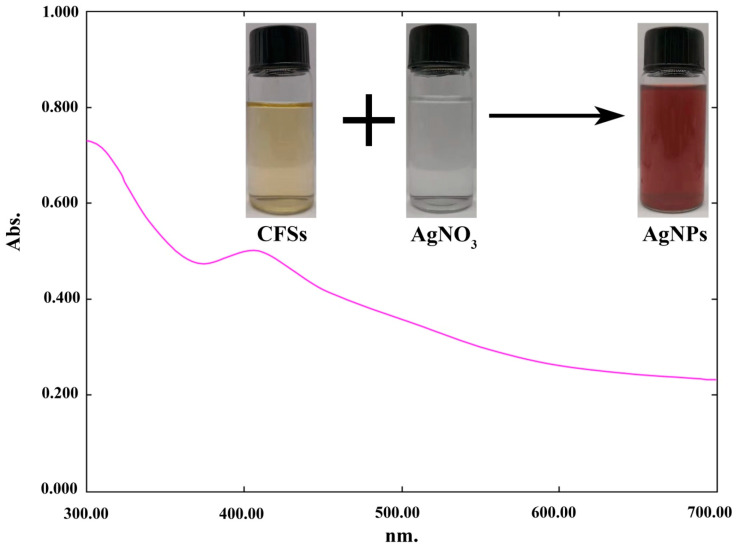
UV-vis absorption spectra of green silver nanoparticles (AgNPs) in the reaction mixture.

Furthermore, the formation of nanoparticles in the mixture was confirmed by a UV spectrophotometer, which showed a spectrum of surface plasmon resonance (SRP) at 412 nm (Figure 2), which is within the range reported earlier [13,28,29]. Similarly, Ahmed et al. [30] confirmed the formation of biogenic AgNPs in the reaction mixture by the presence of the peak at 418 nm.

**Figure 3:**
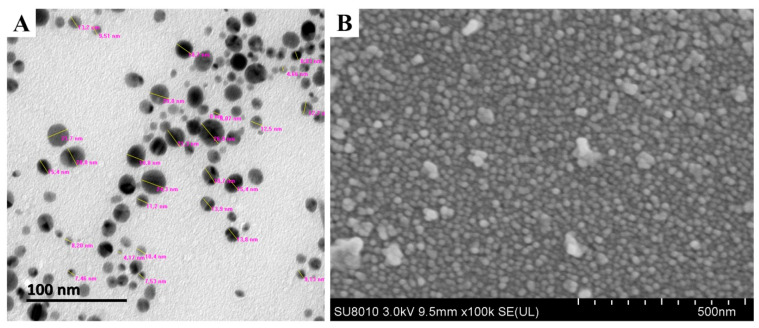
Characterization of AgNPs biosynthesized by using culture filtrates of *B. endophyticus* strain H3 isolated from onion. (**A**) Transmission electron microscopy and (**B**) scanning electron microscopy.

**Figure 4:**
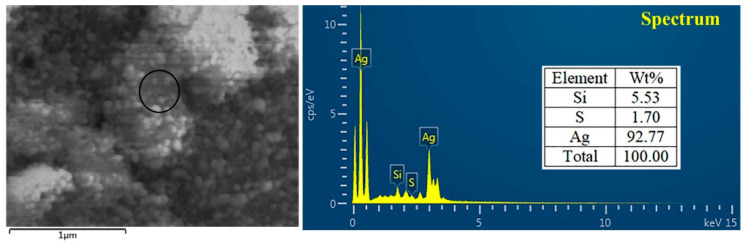
Characterization of biosynthesized AgNPs by using energy dispersive spectrum (EDS).

TEM and SEM observations showed the nanoparticle to have spherical shape with sizes ranging from 4.17 to 26.9 nm in the reaction mixture (Figure 3). The present observations are consistent with the results from previous reports [13,31,32]. The toxicity of AgNPs depends on the variation of particle size. AgNPs have an important influence on fungal cell’s viability and ROS generation in a size-dependent manner. It is evident that the surface area, the volume ratio and the interaction of the surface with the particle size can be changed. Furthermore, sedimentation rate, mass diffusion, binding efficiency and sedimentation rate of NPs on biological or solid surfaces are highly influenced by particle size [33]. For example, Carlson et al. found that the 15 nm AgNPs can produce more ROS compared to 55 nm AgNPs in a macrophage cell line [34]. Furthermore, the EDS result showed that the element peak of silver, silica and sulfur are 92.77, 5.53 and 1.70%, respectively, in the reaction mixture (Figure 4). The results of this study are in agreement with the literature related to silver nanoparticles, where the silver ions peak was confirmed at 3 KeV [30,35].

**Figure 5:**
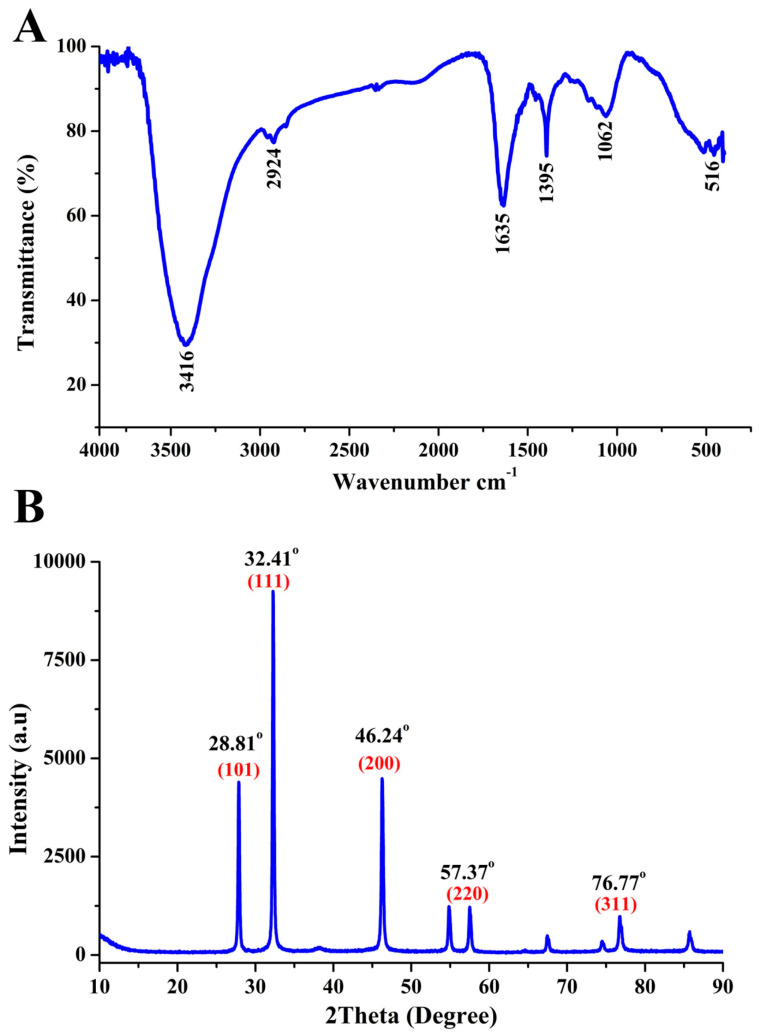
Characterization of the biosynthesized AgNPs. (**A**) Fourier transform infra-red (FTIR) spectra. (**B**) X-ray diffraction (XRD) spectra.

The functional groups of the biosynthesized AgNPs were confirmed by using FTIR analysis. In fact, 6 peaks at 3416, 2924, 1635, 1395, 1062 and 516 cm^−1^ were observed in the FTIR spectra of the synthesized AgNPs, which are shown in (Figure 5A). The main peak of 3416 cm^−1^ is due to the N–H stretching vibrations; a characteristic peak at 1635 cm^−1^ represents C=O carbonyl group and C=C stretching vibrations; the peaks at 2924, 1395 and 1062 cm^−1^ represent the C–H stretching vibrations, C=N bond of Amide II, O–H deformation vibrations, and C–N stretching amine vibrations, respectively; the peak at 516 cm^−1^ represents C–Br stretching. The existence of such groups in the chlorofluorocarbons (CFCs) from endophytic bacterium confirms the presence of proteins and indicates that these functional groups have a major role in reducing Ag^+^ to Ag^0^ [13,32,36]. Similarly, various studies reported the presence of functional groups representing different macromolecules, such as nucleic acids, proteins, lipids, carbohydrates and sugars, surrounding the green NPs which prevents the oxidation and deterioration of nanoparticles [30,37,38].

The crystalline structure of the biogenic AgNPs was determined through the XRD analysis result, that showed five emission peaks of 2θ = 28.81°, 32.41°, 46.24°, 57.37° and 76.77°, compatible with crystalline silver planes (101), (111), (200), (220), (311), respectively (Figure 5B). Similar results have been reported in other studies [11,14,38].

### 3.3. Antifungal Activity of AgNPs

**Figure 6:**
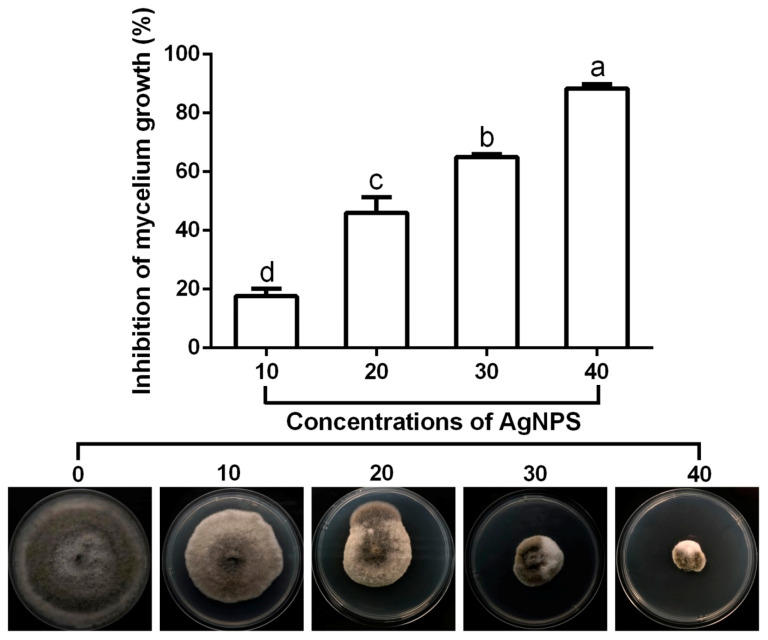
Effect of the biosynthesized AgNPs at four different concentrations (10, 20, 30 and 40 μg/mL) on the mycelial growth of *M. oryaze* strain Gry. The AgNPs at 10, 20, 30 and 40 μg/mL caused an 18%, 49%, 65% and 88% reduction, respectively, in mycelial diameter. Data are a mean value ± standard error of three replicates, and bars with different letters (a–d) are significantly different in LSD test.

The result of antifungal activity revealed that the mycelium growth of *M. oryaze* strain Gry was robustly suppressed by AgNPs, shown in (Figure 6). The inhibitory effect on the growth of mycelium increased with the increase of AgNP concentrations. In fact, AgNPs at 10, 20, 30 and 40 μg/mL caused an 18%, 49%, 65% and 88% reduction, respectively, in mycelial diameter. Similarly, previous studies have shown that AgNPs can be used as an antifungal agent to prevent plants from fungal infection [9,10,39,40,41,42,43]. Although there are different hypotheses available, the antimicrobial mechanisms of AgNPs have not yet been clearly defined. The proposed mechanisms were summarized based on the current literature, as follows: attachment of AgNPs to the surface of the cell membrane, altering the lipid bilayer or increasing permeability of the cell membrane, microbial cell intrusion of AgNPs causing damage to intracellular micro organelles (such as mitochondria, vacuoles and ribosomes) and biomolecules including DNA, protein and lipids, and modulation of the intracellular signal transduction method towards apoptosis [44].

**Figure 7:**
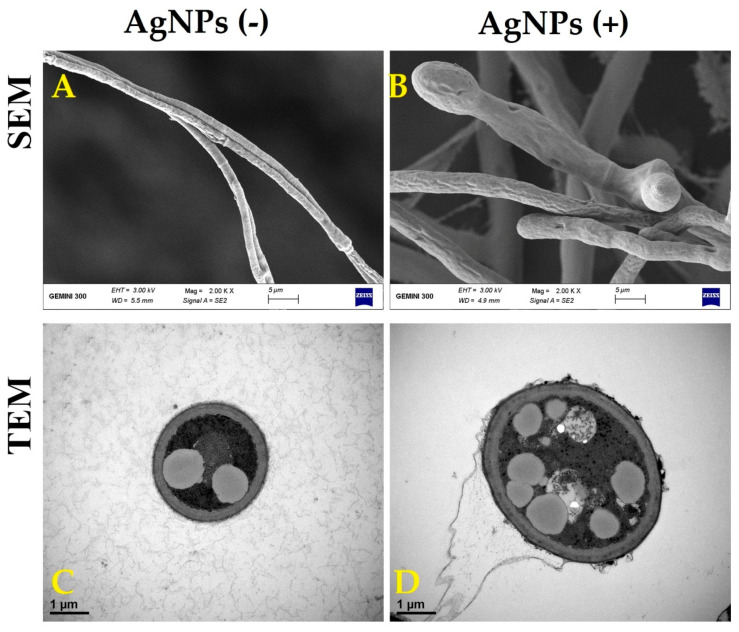
Scanning electron microscopy (SEM) [scale bar = 5 µm] and transmission electron microscopy (TEM) [scale bar = 1 µm] images of *M. oryaze strain Gry* in the absence of the biosynthesized AgNPs; the hyphae showed a normal structural property (**A**,**C**), and in the presence of the biosynthesized AgNPs, it had abnormal structure, swelling and damage to the cell walls’ contents (**B**,**D**).

The fungal cell wall is a flexible structure that performs several functions in determining the shape of the cell. In addition, the cell wall can protect the fungal cells from environmental stresses, such as pH, temperature and changes in osmolality [9,45]. Therefore, interference by causing harm to the fungal cell wall may lead to loss of content and death. In our results, the hyphae of *M. oryzae* strain Gry that were treated with AgNPs showed abnormal structural, swelling and damage to their cell walls causing some loss of contents. In contrast, the cell walls of *M. oryzae* strain Gry had normal structural characteristics in the absence of the AgNPs (Figure 7). Similarly, the antifungal activity of AgNPs and Cu-NPs have been found on the cell walls of many pathogenic fungi such as *Fusarium graminearum*, *Fusarium oxysporum*, *Fusarium solani* and *Colletotrichum gloeoesporioides* [9,45,46].

**Figure 8:**
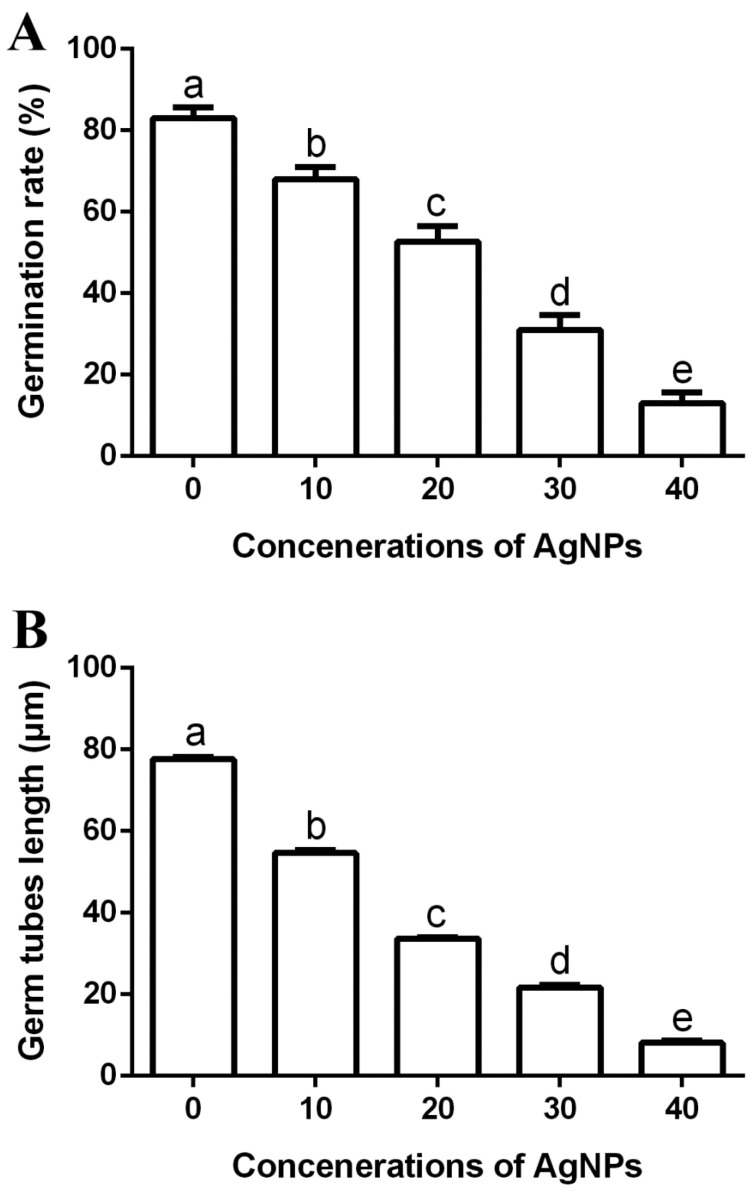
Effect of the biosynthesized AgNPs of four different concentrations (10, 20, 30 and 40 μg/mL) on spores’ germination rate (**A**) and germ tube growth (**B**). Data are a mean value ± standard error of three replicates, and bars with different letters (a–e) are significantly different in LSD test.

The germinated spores of pathogenic fungi are known to perform a major role in colonizing and infecting plants [9,47]. Therefore, inhibition rate of spore germination will significantly reduce the threat of rice fungal pathogens. The biosynthesized AgNPs were able to efficiently inhibit the spore germination and germ tube growth of *M. oryzae* strain Gry, and the antifungal activity increased with the increase in AgNP concentration. In fact, the germination rate of spores was 83%, while the length of the germ tubes was 77.63 μm in the negative control. However, the germination rate of spores was 68%, 53%, 31% and 13%, respectively (Figure 8A), while the germ tubes length was 54.62 μm, 33.73 μm, 21.74 μm and 8.11 μm, respectively, (Figure 8B) in the presence of AgNPs at four different concentrations (10, 20, 30 and 40 μg/mL). Similar results have also been reported in other studies [9,48].

**Figure 9:**
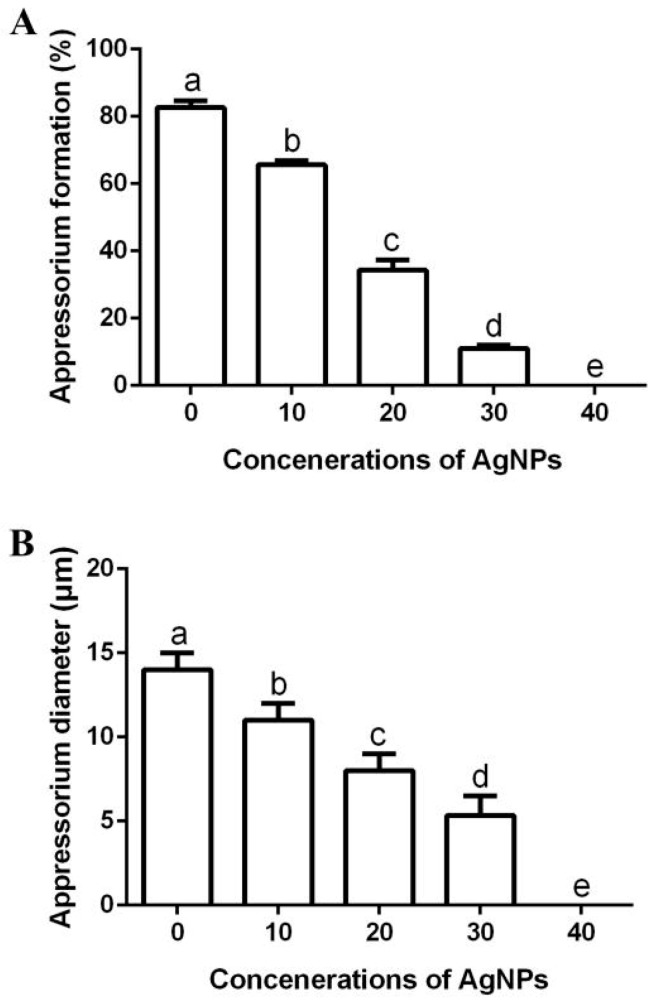
Effect of the biosynthesized AgNPs of four different suspensions (10, 20, 30 and 40 μg/mL) on appressorium formation (**A**) and appressorium diameter (**B**). Data are a mean value ± standard error of three replicates, and bars with different letters (a–e) are significantly different in LSD test.

Many fungi form a specialized infection structure called an appressorium that is necessary and required to penetrate the plant cell walls [1,6]. Therefore, appressorium inhibition significantly reduces the risk of rice fungal pathogens. The biosynthesized AgNPs were able to effectively inhibit the appressorium formation and appressorium diameter of *M. oryzae* strain Gry, while the inhibitory effect increased along with the increase in AgNP concentration. In fact, the appressorium formation rate was 83%, while appressorium diameter was 14.00 μm in the negative control. However, the appressorium formation rate was 66%, 34%, 11% and 0%, respectively (Figure 9A), while the appressorium diameter was 11.00 μm, 8.00 μm, 5.38 μm and 0.00 μm, respectively (Figure 9B) in the presence of AgNPs at different concentrations (10, 20, 30 and 40 μg/mL).

## 4. Conclusions

The green synthesis of AgNPs is a safe alternative to physical and chemical methods. The present study reports for the first time the biosynthesis of AgNPs by using endophytic bacterium isolated from onion. The formation of biogenic AgNPs was further confirmed through UV-vis spectroscopy, FTIR, XRD, SEM, TEM and EDS. In addition, the biosynthesized AgNPs exhibited robust antifungal activity against rice blast pathogen *M. oryaze* strain Gry, which may be mainly attributed to their ability to inhibit spore germination, germ tube growth, appressorium formation and damage of cell well. Overall, these results suggest that biosynthesized AgNPs have the potential to protect rice plants from fungal diseases.
